# Book Review

**Published:** 1897-09

**Authors:** 


**The Medical Environment.** By D. CAMPBELL BLACK, M.D. Pp. 50. Glasgow: Hugh Hopkins. [1896.]—Something of the radical nature of the two addresses here printed as a booklet may be gathered from the fact that the author maintains that hospitals as charitable institutions have now no *raison d'être*, since our poor-law system is, or ought to be, sufficient to meet all cases of destitute sickness. On the subject of hospital reform, which is now attracting so much attention, Prof. Black must be regarded as an able exponent of the views of the malcontents who form an ever increasing army, chiefly no doubt the result of the overcrowded state of the profession. Prof. Black is entirely opposed to conventional usage in medical ethics; but that does not seem quite sufficient reason why he should send out his little book looking so very like an advertisement of a quack doctor. Is it because he does not wish to appear better than the itinerant "professor" who so often announces his medical powers in an unpretentious pamphlet? Those who are interested in the subjects here treated should read these addresses, which, despite their impassioned style and the "animus" which pervades them, have in them much of logic and common sense, and are interesting in that they show what extreme views can be held with regard to these subjects by a man of Prof. Black's standing. Dr. Black, although he regrets the "unworthitess" (*sic*) of these pages, seems anxious that readers should procure some of his other pages; for at the beginning and end of these appear not only announcements of his many publications, but press notices of them, and even opinions extracted from private letters concerning them; and he considerately adds a list of his "miscellaneous contributions," which are too numerous to be all mentioned in the space available.

**The Sweating of the Medical Profession by the Friendly Societies of Australasia, with a practical Scheme for its Abolition.** By LUDWIG BRUCK. Pp. 60. Sydney: Office of the *Australasian Medical Directory and Handbook*. 1896.—It seems to be the fashion in Australia for everybody, indiscriminately, members of Government, ex-ministers of the Crown, pensioners, and land-owners having large incomes, to join friendly societies, with which the country seems to swarm. The Registrar of New South Wales remarks: "Some lodges are fortunate enough to have members so well-to-do that they do not take the ordinary sick pay when invalided, nor even the funeral donation payable on the death of a wife." Such members join only for the benefits of cheap medical attendance and medicine. The rules binding the unfortunate doctors of these societies are of the most tyrannical description. For instance, the Brisbane Associated Friendly Societies provide that in the event of the breach by the doctor of any of the articles hereof such breach shall be reported to the General Committee, who shall have full power, either with or without calling for an explanation from the doctor, to dismiss the doctor from the said service. No appeal shall lie from the decision of the General Committee, and these presents may be pleaded in bar to any action brought by the doctor. So the wretched man is forced even outside the pale of the law! These committees publish annual reports to their subscribers, and endorsed on the back of a report, furnished from the dispensary connected with the Brisbane society, were these remarkable words: "For correcting female irregularities, steel and pennyroyal pills have long been recognised as a safe and efficient remedy. One shilling a box." Given as a piece of general information, this is convincing as a proof that in Australia the morals and the social institutions are equally advanced; further comment is needless. The remedy proposed by the author, for this disgraceful state of things, is singular; some will be inclined to consider it even worse than the disease. He introduces his proposals as follows: "I am well aware that any remedy depending on the unanimity of the profession would be impracticable." That is his opinion of the doctors of Australia, and no doubt he knows them better than we do. So he proposes that every man should start a club of his own, and "admit all classes of society, whether poor or rich, and charge (say) from 1 to 2½ per cent. on their annual income." It does not seem to strike him that people should be made to pay fairly for, and according to whatever services may have been rendered to them; and how he hopes by these means to check professional rivalry and jealousy does not appear. Altogether things in Australia seem to be pretty much as in England, only perhaps a trifle worse.

**A Vest-pocket Medical Dictionary.** By Albert H. Buck, M.D. Pp. vi., 529. London: Baillière, Tindall and Cox. 1897.—As far as the size of the book will allow, this is a very serviceable volume, but in order to accommodate itself to the "vest-pocket," much which should be present of course has to be omitted in the definition and explanation of rare words, although it is a matter for admiration that so much has been got in. There should be a demand for this book, which is the best of these tiny dictionaries we have seen.

**The Progress of Medical Chemistry, comprising its application to Physiology, Pathology, and the Practice of Medicine.** By J. L. W. Thudichum, M.D. Pp. 212. London: Baillière, Tindall and Cox. 1896.—This book is a reprint of a series of papers dealing chiefly with urinary chemistry, and containing a *résumé* of the researches with which the author's name has long been associated. It is interesting as an expression of Dr. Thudichum's views upon many vexed questions, but it also renders very conspicuous the failure of ordinary chemical methods to deal satisfactorily with the complicated products of animal metabolism. The strictly technical matter is interspersed with suggestive, if somewhat pointed, criticism of the work of professors of animal chemistry generally; and the account of the straits to which they have been driven to justify their existence is amusing, and perhaps not altogether devoid of foundation.

**Cycling as a Cause of Heart Disease.** By GEORGE HERSCHELL, M.D. London: Baillière, Tindall and Cox. 1896.—This pamphlet, of 44 pages, gives a wholesome warning against the abuse of the favourite and fashionable pastime of cycling by those who have any cardiac weakness or tendency thereto. There is undoubtedly some danger at present that the craze of the hour may develop into the injury of a lifetime; but the author is himself a cyclist, and as he recognises the utility of the cycle when used in moderation, he does not therefore take an unreasonable view of the situation, and he has no tendency to exaggerate the possible dangers from overstrain of a weak or an already damaged heart. This well-timed injunction against the effects of immoderate cycling is summed up in two chapters,—the one on the dangers incurred by the novice or untrained individual, and the other on the effect of prolonged cycling upon the trained man; and he argues that, when "carried out in such a manner as to keep up a material acceleration of the heart's action or increased blood-pressure for any length of time, it must of necessity sooner or later produce disease of the circulatory apparatus. And this will almost certainly be the case if the rider is *not* a young and healthy adult, but an individual over middle age whose tissues are commencing to undergo degenerative changes."

**Elementary Bandaging and Surgical Dressing.** By WALTER PYE. Revised and in part re-written by G. BELLINGHAM SMITH. Seventh Edition. Pp. viii., 218. Bristol: John Wright & Co. 1896.—This handy volume contains a good deal of useful information packed in small space. It will be much appreciated by those students and nurses who use it as a guide in the earlier months of their professional training. It describes the ordinary methods of applying bandages, splints, and dressings; and in addition has chapters upon the treatment of cases of shock and collapse, of immersion, and of poisoning.

**The Treatment of Wounds, Ulcers, and Abscesses.** By W. WATSON CHEYNE, M.B., F.R.S. Second Edition. Pp. xii., 197. Edinburgh: Young J. Pentland. 1897.—In this edition the author has not in any way modified the methods of treatment which he recommended in the previous edition, of which this appears to be a mere reprint. If surgeons will follow the rules of treatment laid down in this book, which are practically the same as Lister's, they will have at least as good results as can be obtained by aseptic methods, with much less trouble and expense.

**A First Series of Fifty-four Consecutive Ovariotomies, with Fifty-three Recoveries.** By A. C. BUTLER-SMYTHE. Pp. viii., 119. London: J. & A. Churchill. 1897.—It is doubtless a matter of congratulation to have performed a series of fifty ovariotomies without a death, but the mortality of this once dreaded operation has now become so low that one is getting accustomed to such results as that shown by the author. His operative details do not differ from those of most surgeons of repute, and a perusal of the cases themselves does not appear to bring out any new points in the pathology or treatment of these tumours. It is noticeable that two of the cases were followed by insanity, but both recovered. The author has taken commendable trouble to bring the history of his cases up to date.

**Dental Surgery.** By A. W. Barrett, M.B. Third Edition. Pp. xii., 154. London: H. K. Lewis. 1897.—This manual is capitally arranged to facilitate handy reference, and very practical treatment is suggested in every case. It has probably been of great service to medical men in its past editions, and this new one is likely to extend its usefulness.

**Rough Notes on Remedies.** By William Murray, M.D. Second Edition. Pp. viii., 104. London: H. K. Lewis. 1897.—It is only a short time since we noticed the first issue of this book, and the rapid appearance of this second edition is sure proof that the general body of the profession has, with the author, "an undying, nay, an increasingly vital faith in the virtue of medicines." Dr. Murray believes "that much depends upon the method of combining our remedies." The disadvantage of this doctrine lies in the fact that we cannot all claim to have the experience and the good judgment of the veteran.

**Herbal Simples.** By W. T. Fernie, M.D. Second Edition. Pp. xxiii., 651. Bristol: John Wright & Co. 1897.—This edition is very considerably enlarged, and contains much information of a more or less credible kind. The style, however, is diffuse, and the method of composition disjointed. The work has now become a manual of homœopathic treatment by herbal and "simple" remedies; and many such statements as the following are to be found: "Dr. Burnett has lately taught (1895) that a too free use of Cloves will bring on albuminuria; and that when this disease has supervened from other causes, the dilute tincture of Cloves, third decimal strength, will frequently do much to lessen the quantity of albumen excreted by the kidneys. From five to ten drops of this tincture should be given with water three times a day." Some of the statements are curious, *e.g.*, "many quarts of cooked garden snails are sold every week to the labouring classes in Bristol;" and furthermore, some other statements are astonishingly erroneous, *e.g.*, "The special qualities of the Pimento reside in the rind of [the] berries; and this tree is the *Bromelia ananas*"! The work does not call for detailed criticism in the pages of this Journal.

**What to Do in Cases of Poisoning.** By William Murrell, M.D. Eighth Edition. Pp. 290. London: H. K. Lewis.

1897.—That another edition should be required in rather more than three years, is a proof that this little work is fairly well appreciated. As the author states that it "has been thoroughly overhauled and revised" for the present edition, we expected to find no errors, however insignificant. There is a great improvement in one respect, we have now an index of 10 pages. The statement that conium is "common in all our hedges" is by no means true; umbelliferous plants are—but not conium. We have still "Cytisis Laburnam" for "Cytisus Laburnum" (p. 162), and (p. 187) "decrotism" for "dicrotism." Under nitric acid poisoning we have still not a word about the fumes being fatal when inhaled in any quantity. Ice-creams are looked upon with the direst suspicion, and justly so. "Whereas in good drinking water there are rarely more than 100 bacteria per cubic centimetre, three samples of ice cream which were analysed contained respectively 2,150,000, 4,200,000, and 5,340,000 bacteria."

**The Private Sanatoria for Consumptives, and the Treatment adopted within them.** By ARTHUR VON JARUNTOWSKY. Translated by E. CLIFFORD BEALE, M.B. Pp. 48. London: The Rebman Publishing Company, Limited. [N.D.].—The largest and most important of the sanatoria for consumptives in Germany and Switzerland are here described. None claims absolute perfection, but if "it were possible to unite the situation, the surroundings, and above all, the Park of Goerbersdorf with the internal fittings of the Hohenhonnef Sanatorium, and the dietetic method employed at Falkenstein, then, for the first time, we should have a Sanatorium for Consumptives which would approach very near to the Ideal." From an analysis of 6,000 cases emanating from Goerbersdorf and Falkenstein it appears that about 25 per cent. of the cases are reported as cures. But surely we have advanced somewhat since the time of Hippocrates, who first recommended suitable climatic conditions and good living. We cannot accept the dictum that "the treatment of Consumption with drugs has gone into utter bankruptcy." Much is to be said in favour of sanatoria for consumptives, but why is it that drugs seem to be unknown in them? Surely a judicious combination of dietetic, climatic, and medicinal treatment is likely to give better results than either by itself. The translator observes that "no more suitable climate exists for Consumptives than that of a fine English Summer," and much may yet be done towards the establishment of English sanatoria which may possess the most essential of the climatic requirements and conditions demanded by Brehmer and other pioneers of sanatorium treatment.

**The Climate of Bournemouth in relation to Disease, especially Phthisis.** By A. KINSEY-MORGAN, M.D. Pp. 51. Bristol: John Wright & Co. 1897.—The author of this little essay gives many good reasons why phthisical patients should resort to

Bournemouth. (1.) *Dryness of soil.* Its situation on the middle eocene strata gives it a porous sandy soil, far removed from the underlying and impervious beds of London clay of the lower eocene. "On the east side of the town the sands extend to the river at Christchurch, and no clays whatever come within . . . fifty feet of the surface." (2.) *Purity of air.* This is added to by "the beneficial inflence of the pine forests, which surround it, and of the groves of pine trees, with which it is interspersed." (3.) *Sanitary conditions.* These are claimed to be in advance of those of many foreign health resorts. (4.) *Meteorological conditions.* The temperature from November to February is about two degrees higher than at Greenwich. The average number of hours of bright sunshine annually registered is 1570 as contrasted with 1365 at Kew; the longest possible period of sunshine on the 1st of January in the principal Swiss health resorts is six hours and ten minutes, while at Bournemouth it is seven hours and fifty-three minutes. (5.) *Morbific influences.* Dr. Kinsey-Morgan says, "Direct infection may sometimes take place, but I may state that in Bournemouth I have never yet been able to substantiate this theory." The conditions described give Bournemouth an especial capacity for improving debilitated constitutions generally, and pulmonary diseases in particular. Convalescents from lung trouble soon lose the vestiges of disease, bronchial *râles* and delayed repair soon become things of the past. Here the invalid will find good hotels, every comfort, fresh scenes, and new associations. What more can he wish for, or where better can he get it? The book is well padded with illustrative cases, and we notice that drugs take a very secondary rank in Bournemouth treatment.

**Cornwall as a Winter Resort.** Third Edition. Revised by E. Kitto. Pp. 23. Plymouth: W. Brendon & Son. 1896.—The recent boom of Falmouth by Sir Joseph Fayrer has directed the attention of the invalid in search of winter quarters to that part of the British Islands where the rhododendrons and camellias flower throughout the whole winter in the open air. The climatic conditions of the county are as agreeable to the healthy as they are a necessity to the weak and the bronchitic *poitrinaire.* They are well set forth in this pamphlet, which, in its present form, has been revised by the Superintendent of Falmouth Observatory and issued apparently as a pleasing advertisement of the Great Western Railway Company.

**Guy's Hospital Reports.** Vol. LII. London: J. & A. Churchill. 1896.—The principal article in this volume is an obituary notice of Arthur Edward Durham, by Mr. Jacobson, who writes with the enthusiasm of an old friend and pupil a most appreciative article on this distinguished surgeon of Guy's Hospital. Mr. Jacobson's writing is adorned with much literary grace and polish; and this article will be read with delight by the many old students of Guy's, who look back with the happiest recollections to the kindly though erratic genius of Arthur Durham, whose portrait forms the frontispiece to the volume. Among the other contents of the present issue are a paper on "Hallux Flexus, Claw Toe, and Pes Cavus," by Mr. N. Davies-Colley; "Cases of Cirrhosis of the Liver in Children, with some Remarks on Cirrhosis," by Dr. Frederick Taylor; and a paper on "Laceration of the Female Perinæum, its Varieties, Mechanism of Causation, and Treatment," by Dr. Thos. G. Stevens.

**Transactions of the Association of American Physicians.** Vol. XI. Philadelphia: Printed for the Association. 1896.—This volume, of nearly five hundred pages, contains papers from the leaders of the profession in America. The Association, "being the scientific representative of internal medicine in America, ought to be recognized all over the Union as the scientific lawgiver." (Dr. A. Jacobi.) What the New York Academy of Medicine is calculated to become for New York city this Association ought to be for the Union, and beyond it, through the scientific labours of its members. The Association sets a high aim before itself, and we are bound to admit that the papers read fully justify the standard. Amongst the subjects discussed are the following: leukomaïn-poisoning, diphtheria antitoxin, parasitic chyluria, prevalence and fatality of pneumonia, œsophageal hemorrhage, subphrenic abscess, terminal infections, virulence of diphtheria bacilli, prognosis in pneumonia, mescal buttons, actinomycosis, pathogenic spirilla, and X-rays. It will be seen that the book is more suitable for study than for review.

**Transactions of the Medical Association of the State of Alabama. The State Board of Health.** Montgomery: Brown Printing Co. 1896.—On looking through the statistics in the various parts of the book, we are amazed at the frightful death-rates at some of the convict establishments: in 1895 at Coalburg the death-rate was 102 per 1000, and at Pratt Mines it was 105 per 1000; it is certainly to be hoped that before this a very marked improvement has taken place. There is a most excellent suggestion in the annual message of the President, and one that would work well in this country; it is with regard to the question of insanity in murder cases: the President suggests that a board of three or five of the most competent physicians in the State should be appointed by the State, and that they should decide the question of sanity in any case where that question came up. The volume is well printed and contains a large quantity of useful information; but too much space is given to the Roll of the County Societies, which form the State Association, and that which is styled an "Index" should be called a "Table of Contents."

**Transactions of the New Hampshire Medical Society.** Concord: The Republican Press Association. 1896.—This interesting volume contains a complete account of the proceedings at the one hundred and fifth anniversary of this Society, which is one of the oldest in the world, and now contains nearly 300 members. An unusual custom, and one which, in our opinion, might sometimes with advantage be copied in kindred meetings, is the reporting of the amusing after-dinner speeches, or "post-prandial exercises" as they are called, at the annual dinner. "If we know we are to be reported, we will be more apt to array our words in their Sunday clothes; will be more choice in our language," says the chairman at the dinner, Dr. A. Noel Smith. In another speech a witty parson, who had consulted his cyclopædia, traces the evolution of specialism from the prehistoric times when the powers of the lawyer, the priest, and the physician were vested in one individual. The book contains the president's address, and reports on medicine, surgery, variola, medical legislation, and other subjects, with the remarks *in extenso* of those members who spoke. The discussion that followed Dr. John W. Staples's communication on physical exercises and athletics is well worth reading. Dr. Staples ably advocates the great advantage that accrues to the individual, physically, mentally, and morally, from athletic exercises. He believes the proper expansion of the lung, that these exercises and the training for them necessitates, is to a great extent a safeguard against inflammatory changes, and therefore against the development of tuberculosis. Both in boys and girls there has been a better physical development during the past decade, and this is due to the greater prominence that has been given in schools to games and athletic contests. A few subsequent speakers were inclined to the opinion that athletics may be and often are overdone, and many different opinions were expressed as to the relative value of gymnastics, games, and bicycling, one speaker holding that the latter exercise is very beneficial even in cases of diseased heart, provided that the learning to ride and that the practice of riding is gradual. We are inclined to agree that bicycling is one of the healthiest pastimes, especially as the amount of effort necessary is almost entirely under the control of the individual rider. Other papers in the book are well worth study.

**Transactions of the South Carolina Medical Association.** Charleston: Walker, Evans and Cogswell Co. 1896.—To read the papers published in this volume is to become convinced that the practitioners of South Carolina are well abreast of the times. Typographical errors however are not uncommon, and much of the phraseology is "loose," giving one the impression that the book was printed without sufficient supervision.

**Transactions of the Medical Society of London.** Vol. XIX. London: Harrison and Sons. 1896.—One hundred and forty pages in this volume are occupied by Mr. Watson Cheyne's very valuable Lettsomian lectures on the objects and limits of operations for cancer, which, in their reprinted form, have been already reviewed in this Journal. The volume also contains many interesting records of abdominal surgery, particularly of cases of enterectomy for malignant growth; and the papers on extra-uterine gestation, together with a full account of the discussion which followed, provide much instructive reading. The diagnosis and treatment of early cancer and cysts in the breast have been ably discussed by Mr. Bryant. An interesting paper by Mr. Edmund Owen, on Cleft Palate, called forth a valuable discussion on the subject, which is reported at length. Mr. Lockwood contributes an important paper on the Operation for Radical Cure of Hydrocele by Excision of the Sac. There are many other important contributions in this volume, which is almost entirely a record of surgical work, and is, perhaps, some evidence of the present trend of practice which seems likely to result in our finding every portion of the body invaded by the operator, whose appetite for conquest has been made keener by the revelations of the X rays.

**Reports and Papers of the American Public Health Association.** Vol. XXI. Concord: Republican Press Association. 1896.—This handsome volume, of over 400 pages, contains an immense variety of material on subjects of interest in almost every branch of public health. There is much value in the opportunity for comparison between modes of thought and action in other countries and our own; and the present volume may be consulted with not only interest but profit.

**Transactions of the Epidemiological Society of London.** Vol. XV. London: Shaw and Sons. 1896.—We notice as of special local interest Dr. Dowson's paper on "Diphtheria in Older and Newer Bristol," and this obscure disease receives further consideration in Dr. Goodall's "Post-Scarlatinal Diphtheria in the Hospitals of the Metropolitan Asylums Board," and in Dr. Geo. Reid's "Infectious Sore-Throat and Diphtheria." The interesting and important question of "return" cases of scarlatina is adequately dealt with in a paper by the late Mr. T. W. Thompson, of whom the later pages of the volume contain an appreciative "In Memoriam." The other articles fully maintain the valuable character of the Society's Transactions.

**The Edinburgh Medical Journal.** New Series. Vol. I. Edinburgh: Young J. Pentland. 1897.—This, the first of the new series, forms a handsome volume of nearly seven hundred well-printed pages, on a variety of interesting topics. Dr. George Buchanan's retrospect of fifty years' experience of anæsthesia—first as student; next, assistant; and then, hospital surgeon—is a fitting commencement to a volume published in this great Jubilee year. We offer our congratulations to Dr. Gibson on the visible success of his editorial work, and to the publisher on the attractive appearance of the volume.

**Formulaire des Médicaments Nouveaux.** Par H. Bocquillon-Limousin. 7e édition. Paris: J.-B. Baillière et Fils. 1896. Avec Supplément pour 1897.—An interesting and useful account of many drugs is given in this book of 338 pages. A large number of these preparations have yet to make their reputation; they may be an improvement on their predecessors, but of many it is safe to predict that they will soon be replaced by new ones or rejected for old ones. The newest is not always the best. Dr. Huchard, who writes an introduction to the book, says: "Il est non seulement utile, mais indispensable, à la fois aux chercheurs, aux praticiens et aux élèves."

**Burdett's Hospitals and Charities.** By Henry C. Burdett. London: The Scientific Press (Limited.) 1897.—We have on previous occasions expressed a favourable opinion of this useful annual, and the volume for this year does not fall below previous issues in value and interest. The mass of statistics contained prevents any criticism beyond a general one; though there is the usual opening chapter of comment, expressing the opinions of H. C. Burdett. There is the proper amount of Jubilee notice, and some sanguine expressions of hope about the Prince of Wales's Fund which do not appear likely to be realised. We may take this opportunity of congratulating Sir Henry on his K.C.B., which we think he thoroughly deserves for the indefatigable zeal and interest he has shown on behalf of the hospitals of the world. "Dr." Barnardo comes in for his annual wigging, but there is less than usual about the Radcliffe Infirmary.

**Year-Book of the Scientific and Learned Societies of Great Britain and Ireland.** London: Charles Griffin and Company, Limited. 1897.—The information given in this book is useful, and so far as our local Society is concerned is fairly correct; but we do not like the capricious insertion and omission of the initials of Christian names. We must repeat that its value as a work of reference would be increased if the index included the titles of papers and the names of authors of such papers.

---

